# Schizandrin A exhibits potent anticancer activity in colorectal cancer cells by inhibiting heat shock factor 1

**DOI:** 10.1042/BSR20200203

**Published:** 2020-03-12

**Authors:** Bing-chen Chen, Shi-liang Tu, Bo-an Zheng, Quan-jin Dong, Zi-ang Wan, Qiao-qiong Dai

**Affiliations:** The Surgical Department of Coloproctology, Zhejiang Provincial People’s Hospital, Hangzhou 310014, Zhejiang Province, China

**Keywords:** CRC, HSF1, molecular docking and molecular dynamic simulation, Sch A

## Abstract

Heat shock factor 1 (HSF1) is a powerful multifaceted oncogenic modifier that plays a role in maintaining the protein balance of cancer cells under various stresses. In recent studies, there have been reports of increased expression of HSF1 in colorectal cancer (CRC) cells, and the depletion of the HSF1 gene knockdown has inhibited colon cancer growth both *in vivo* and *in vitro*. Therefore, HSF1 is a promising target for colon cancer treatment and chemoprevention. In the present study, we found that Schizandrin A (Sch A) significantly inhibited the growth of CRC cell lines by inducing cell cycle arrest, apoptosis and death. Through HSE luciferase reporter assay and quantitative PCR (qPCR), we identified Sch A as a novel HSF1 inhibitor. In addition, Sch A could effectively inhibit the induction of HSF1 target proteins such as heat-shock protein (HSP) 70 (HSP70) and HSP27, whether in heat shock or normal temperature culture. In the Surface Plasmon Resonance (SPR) experiment, Sch A showed moderate affinity with HSF1, further confirming that Sch A might be a direct HSF1 inhibitor. The molecular docking and molecular dynamic simulation results of HSF1/Sch A suggested that Sch A formed key hydrogen bond and hydrophobic interactions with HSF1, which may contribute to its potent HSF1 inhibition. These findings provide clues for the design of novel HSF1 inhibitors and drug candidates for colon cancer treatment.

## Introduction

Colorectal cancer (CRC) is the third most common cancer and the second most common cause of cancer-related deaths worldwide [1]. According to the GLOBOCAN 2018 estimates of cancer morbidity and mortality, it is estimated that more than 1.8 million new CRC cases have been diagnosed in 2018 and over 880000 patients have died from CRC, accounting for approximately 10% of cancer morbidity and mortality [2]. Among all CRC cases, 75% of them are diagnosed at an early and localized stage, while the remaining patients have metastatic disease [3]. For these patients with unresectable CRC, the main method of drug therapy remains the use of chemotherapy regimens, such as 5-fluorouracil plus oxaliplatin or irinotecan (FOLFOX/FOLFIRI) [4]. In recent decades, the use of targeted therapies including anti-EGFR agents (cetuximab and panitumumab) or antiangiogenic molecules (bevacizumab and aflibercept) in association with cytotoxic drugs has dramatically improved the survival rate of patients [5]. Unfortunately, after a few months of treatment, many patients inevitably develop acquired drug resistance, which greatly limits the clinical effectiveness of these targeted therapies [6,7]. Therefore, it is urgent to discover more novel candidate targets for the clinical treatment of CRC.

Heat shock factor 1 (HSF1) is the master regulator of the proteotoxic stress response (PSR) in all eukaryotes and cancer cells [8]. Through the induction of various molecular chaperones-heat-shock proteins (HSPs), HSF1 maintains cellular proteostasis through facilitation of the folding, transportation, ubiquitination, and proteasomal degradation of proteins [9]. A growing number of studies have revealed that HSF1 is overexpressed and structurally activated in multiple cancerous cells, such as CRC, hepatocellular carcinoma, pancreatic cancer, and so on [10,11]. The strong ability of HSF1 and its mediated PSR to guard cellular proteostasis under stress conditions is critical for the survival, proliferation, metastasis, and development of drug resistance of cancer cells [12]. Besides, some recent findings show that HSF1 could drive a pro-oncogenic transcriptional program to promote tumorigenesis far beyond its classical function in protein homeostasis [13,14]. Taken together, the HSF1 signaling networks constitute an intricate and pivotal pro-oncogenic pathway, and HSF1 inhibitors have become a hotspot in molecular targeted cancer therapy research [15].

In recent years, increasing evidence has demonstrated that the discovery of novel bioactive molecules derived from natural medicinal plants could be a feasible and efficient way for the research and development of anticancer drugs [16]. Dibenzocyclooctadiene lignans is a class of multifunctional small molecules isolated from the fruit of *Schisandra chinensis (Turcz.) Baill* (SC), which has attracted widespread research attention worldwide [17]. Schizandrin A (Sch A) is one of the most important bioactive dibenzocyclooctadiene lignans. Sch A, also commonly called as deoxyschizandrin, has long been used in traditional Chinese medicine to treat a variety of diseases such as spontaneous sweating and chronic asthma. The latest research on Sch A reveals that Sch A has a good growth inhibitory effect on human breast and ovarian cancer cells, and that its antitumor molecular mechanism may be related to ER stress, Wnt/β-catenin and PI3K/Akt signaling pathways [18,19]. Moreover, researchers have discovered that Sch A even exhibits great potential in reversing multidrug resistance (MDR) of breast cancer and colon cancer cells. However, whether and how Sch A influences the progress of CRC has never been fully evaluated.

In the present study, the anticancer activity of Sch A was initially tested on four CRC cell lines. MTS, clone formation and flow cytometry results showed that Sch A markedly induced the growth retardation, cell cycle arrest, and apoptosis of CRC cells. Furthermore, Western blot and Surface Plasmon Resonance (SPR) assays demonstrated that the anti-CRC effects of Sch A might be mediated through the direct interaction with HSF1 to suppress the expression of HSF1 downstream pro-oncogenic genes. Computer simulations further indicated that Sch A bound to several key residues of HSF1 mostly through hydrogen bonding and hydrophobic interactions.

## Materials and methods

### Cell culture and reagents

CRC cell lines DLD1, RKO, SW480, SW620 and normal human colon epithelial cell line CCD 841 CoN were obtained from Shanghai Institute of Biosciences and Cell Resources Center (Chinese Academy of Sciences, Shanghai, China). The cells were routinely cultured in RPMI-1640 or DMEM (Gibco/BRL lifeTechnologies, Eggenstein, Germany) supplemented with 10% fetal bovine serum (FBS; HyClone, Logan, UT), 1% penicillin/streptomycin solution in a humidified atmosphere of 5% CO_2_ at 37°C. The primary antibodies used in the present study were all purchased from Cell Signaling Technology (Danvers, MA), including HSF1, HSP70, HSP27, Bcl-2, Cleaved poly ADP-ribose polymerase (PARP), Cleaved-caspase-3, p-Rb (S807/811), Cyclin D1, Cdk4, Cdk6, and β-Actin. The Bcl-2 antibody was procured from Abcam (Cambridge, U.K.). Goat anti-rabbit IgG-horseradish peroxidase (HRP) secondary antibody was obtained from Santa Cruz Biotechnology (Santa Cruz, CA); human recombinant HSF1 full-length protein was purchased from Abcam (Cambridge, U.K.), and their purities were both more than 85% assessed by SDS/PAGE. The palindromic 4× HSE reporter and all primers used in the present study were provided by TSINGKE biological Technology (Beijing, China), Sch A was purchased from Medchem Express (Shanghai, China), and its purity was greater than 99% detected by high performance liquid chromatography.

### Cell viability assay

The Cell Titer 96 Aqueous Non-Radioactive Cell Proliferation Assay Kit (Promega, U.S.A.) was utilized to measure cell viability. RKO, DLD-1 cells (5 × 10^3^ cells/well) and SW620, SW480 (6 × 10^3^ cells/well) were seeded in 96-well plates overnight. This was followed by the removal of the culture medium and the exposure of all cells to different concentrations of chemical treatment for 72 h. The absorbance value of each well was determined by a microplate reader at 490 nm. Each experiment was repeated for three times.

### Clonogenic assay

RKO cells were seeded at a density of 800 cells per well in six-well cell culture plate overnight. Then Sch A was mixed into the culture medium and added at different concentrations. After 24 h, the culture medium was replaced with fresh culture medium, and was allowed to grow for an additional 14 ays. Following this, the medium was removed from the colonies, which were then washed twice with PBS, fixed with methanol for 15 min, stained with Crystal Violet for 15 min, and finally washed with running water for 5 min. Colonies containing more than 50 cells were counted, and then photographed for visualization.

### Cell apoptosis analysis

Flow cytometer analysis and Western blotting were used to detect cell apoptosis. For flow cytometer analysis of apoptosis, cells were treated with trypsin for 24 h, washed twice with PBS, and re-suspended in 100 μl of 1 × binding buffer. Five microliters of FITC Annexin V and propidium iodide (PI) (556547, BD Biosciences, U.S.A.) were added to the cell suspension and then incubated at room temperature for 15 min. After dilution with 400 μl binding buffer, the samples were analyzed using an ACS Calibur flow cytometer (BD). For apoptosis using Western blotting, cleaved caspase 3, cleavage of PARP, Bcl-2, and BCl-xL were analyzed.

### Cell cycle analysis

Cells (3 × 10^5^ cells/well) were seeded in six-well plates and allowed to adhere overnight. The next day, the cells were treated with different compounds as indicated for 24 h. The cells were then trypsinized, washed, and fixed in 75% ice-cold ethanol at 4°C overnight. After centrifugation, the pellets were washed with cold PBS, suspended in 500 μl PBS with 50 mg/ml PI and incubated in the dark at 4°C for 30 min. Then cell suspension was tested using an FACS Calibur instrument (Becton Dickinson FACSCalibor, BD Biosciences, Franklin Lakes, NJ, U.S.A.).

### Western blotting

After 24 h of treatment as indicated, the cells were washed once with PBS. The total proteins were extracted with RIPA buffer supplemented with protease inhibitors (Roche, U.S.A.) and quantified with BSA standard methodology. Equal amounts of proteins were separated by SDS/PAGE and transferred to a PVDF membrane. After blocking with 5% non-fat dry milk in TBST for 1.5 h, the membrane was incubated with a specific primary antibody diluted 1:1000 overnight at 4°C, and the secondary antibody was then conjugated with HRP (1:5000, Santa Cruz, CA) at room temperature for 2 h. The immunoreactive bands were visualized with enhanced chemiluminescence liquid (Millipore, Billerica, MA, U.S.A.) in Amersham Imager 600 system (GE Healthcare Life Sciences, Shanghai, China).

### Quantitative real-time PCR

Total RNA was extracted with TRIzol reagent (Invitrogen) following the manufacturer’s protocol. RNA concentrations were quantified by NanoDrop 2000 (Nanodrop, U.S.A.). Two micrograms RNA was reverse transcribed with High-Capacity cDNA Reverse Transcription Kit (Applied Biosystems, U.S.A.). Quantitative real-time PCR was performed to determine the mRNA expression level using SYBR Green Master Mix Kit and Light Cycler 480 II system (Roche, Shanghai, China).

The following primers were used for RT-PCR:

HSP70 forward primer, 5′-GTGCATTGCAGTGTGCCATC-3′, and HSP70 reverse primer, 5′-GGGCAAATCCTGAGGAGAGC-3′;

HSP90 forward primer, 5′- TGCGGTCACTTAGCCAAGATG-3′, and HSP90 reverse primer, 5′-GAAAGGCGAACGTCTCAACCT-3′;

HSP27 forward primer, 5′-GGCATTTCTGGATGTGAGCC-3′, and HSPA6 reverse primer, 5′-AGCAGGCAGGACATAGGTGC-3′;

ACTB forward primer, 5′-ACTCTTCCAGCCTTCCTTCC-3′, and ACTB reverse primer, 5′-CCATGCCAGTGAGCTTCCCGTTC-3′.

Relative quantities of HSP70, HSP90 and HSP27 mRNA were normalized against ACTB mRNA.

### SPR analysis

SPR experiments were performed on a ProteOn XPR36 Protein Interaction Array system (Bio-Rad Laboratories, Hercules, CA, U.S.A.). Briefly, HSF1 solution in PBST (5 mM, pH 7.4) at a concentration of 1 mg/ml was diluted to 30 μg/ml with sodium acetate buffer (pH 4.5). The chip was activated with EDC/NHS (10 μl/min for 600 s). Then, HSF1 was loaded (5 μl/min for 400 s) and immobilized covalently. Approximately 7000 RU of HSF1 was immobilized on the chip. Any excess of unbound HSF1 was removed by flowing PBS solution (5 mM, pH 7.4, with 5%, w/v, DMSO). Sch A was prepared as 50–200 μM solution in PBS solution (5 mM, pH 7.4, with 5%, w/v, DMSO), and injected (10 μl/min for 100 s). Five concentrations were injected simultaneously at a flow rate of 30 μM/min for 120 s of association phase, followed with 120 s of dissociation phase at 25°C. The final graph was obtained by subtracting blank sensorgrams from the duplex or quadruplex sensorgrams. Data were analyzed using the ProteOn manager software.

### Luciferase reporter assay

The double-strand DNA containing four copies of the palindromic HSE (5′-GATCTAGAACGTTCTAGAACGTTCTAGAACGTTCTA-3′) was synthesized and inserted into the multiple cloning site (MCS) of pGL3-basic vector (Promega, U.S.A.), which was named as p(HSE)_4_-basic vector. Then, the Dual-Glo® Luciferase Assay System (Promega, U.S.A.) was used to measure the activity of the reporter. Briefly, RKO cells (3 × 10^5^ cells/well) were seeded in six-well plates. After being cultured overnight, cells were co-transfected with 2 μg of p(HSE)_4_-basic vector and 0.2 μg of pRL-TK using X-treme GENE HP DNA Transfection Reagent (Roche, U.S.A.) according to the manufacturer’s protocol. The *Renilla* luciferase gene was transfected as an internal control vector. Twenty-four hours after transfection, cells were trypsinized and seeded in 12-well plates at a density of 8 × 10^4^ cells per well. The next day, cells were incubated with Sch A at different concentrations for 30 min, exposed to heat shock at 43°C for 60 min, and then incubated further at 37°C for 5 h. Firefly and *Renilla* luciferase activities were measured using a dual-light reporter gene assay kit (Promega) with a Synergy 2 BioTek plate reader (BioTek, U.S.A.).

### Molecular dynamics simulation of the complex between HSF-1 and Sch A

The crystal structure of HSF-1 was downloaded from the Protein Data Bank database (code: 5D5U), and then processed using the *UCSF Chimera* software [20,21]. Then, the *AutoDock* 4.2.6 software was employed to predict the binding mode between HSF-1 and Sch A [22]. The binding site was predicted by *Caver* Web server, and covered by a grid box size of 22.5 × 22.5 × 22.5 Å dimensions [23]. The globe conformational sampling was set to trials of 200 dockings, and settings were set as default. The optimal complex with lowest binding free energy was chosen for molecular dynamics (MD) simulation analysis.

The restrained electrostatic potential (RESP) method based on the HF/6-13G* basis set was employed to compute the partial atomic charges for Sch A. Then, the ff14SB force field and General Amber Force Field (GAFF) were assigned to HSF-1 and Sch A via *LEaP* module in *Amber 18* program, respectively [24,25]. Thereafter, the complex was solvated in a water box with the TIP3P model extended 15 Å distance between the complex surface and the water box boundary. In addition, chloride ions were used to maintain electron neutrality.

Before performing productive MD simulations, initial minimization, heating and equilibration were employed. At first, the simulated system was submitted to a 15000-step steepest descent algorithm and a 15000-step conjugated gradient algorithm to properly redirect the water molecules and the bad contacts of the entire system. The simulation system was then heated from 0 to 310 K in 300 ps. Subsequently, the simulated system was equilibrated in the isothermal isobaric (NPT) ensemble with 1 ns. Finally, the simulated system was submitted to production MD for 500 ns in the NPT ensemble. During the productive MD simulations, Particle Mesh Ewald (PME) method and the non-bonded cut-off distance was set to 10.0 Å to handle the long-range electrostatic interactions and non-bonding interactions. The temperature and pressure were set to 310 K and 1 atm, respectively [26,27]. Coordinates were saved every 10 ps for numerical analysis using the *CPPTRAJ* module in the *AmberTools 18* program [28]. Five hundred snapshots extracted from the equilibrated trajectory from 400 to 500 ns were utilized to calculate binding free energy calculations and analysis key residues using Molecular Mechanics/Generalized Born Surface Area (MM/GBSA) method [29,30]. During MM/GBSA calculations, a modified Generalized Born model (GB^OBC1^) developed by Onufriev et al. [31] was applied. The conformational entropy was not calculated because of high computational cost and low prediction accuracy [32]. Other parameters were set as default.

### Statistical analysis

The results are presented as means ± standard errors (S.E.M.). Data analysis was performed using one-way ANOVA in GraphPad Pro (GraphPad, San Diego, CA, U.S.A.). The results were statistically significant at *P*<0.05. All the experiments were repeated for a minimum of three times.

## Results

### Sch A suppresses CRC cells proliferation and clone formation

**Figure 1:**
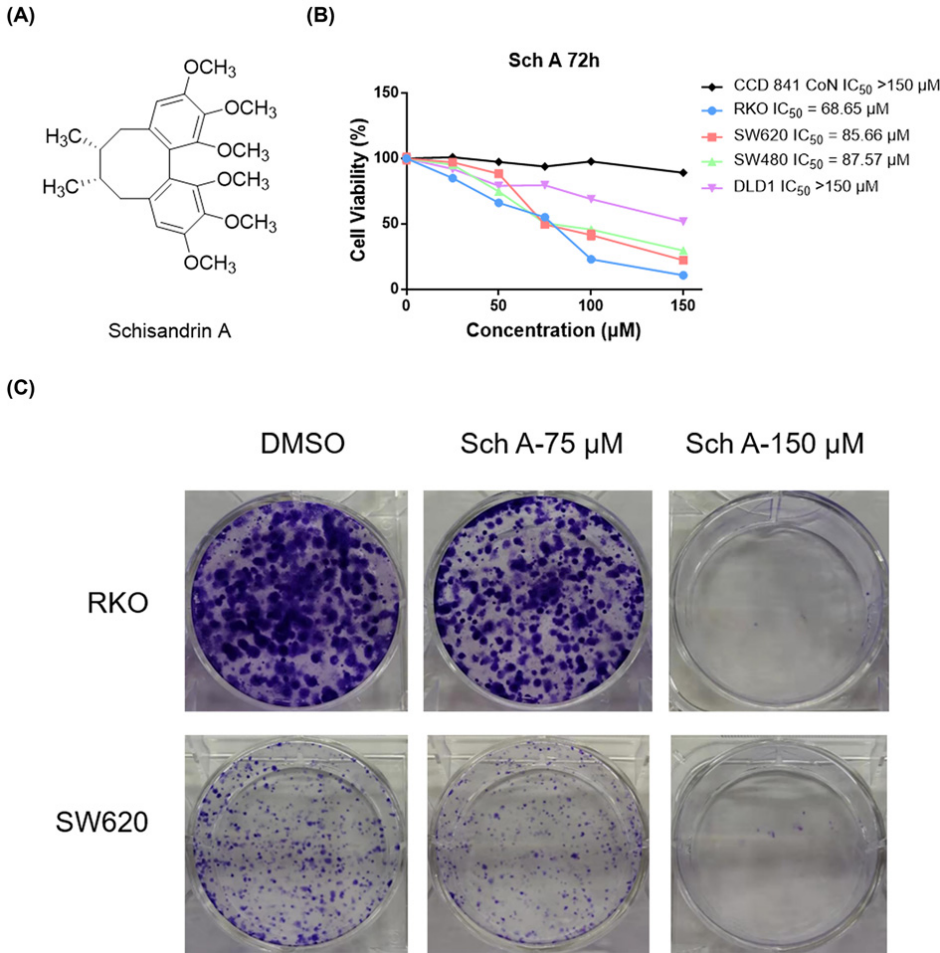
Sch A is a novel anti-growth natural-derived compound against CRC cells (**A**) Chemical structure of Sch A. (**B**) Antiproliferative effects of Sch A on CCD 841CoN, RKO, SW620, SW480 and DLD-1 cells. Cells were treated with the indicated compounds at different concentrations (150, 125, 100, 75, and 50 μM) for 72 h. Subsequently, cell viability in each group was detected by MTS assay. The data were obtained from three to five independent experiments. (**C**) Effect of Sch A on RKO and SW620 cells clone formation. Cells were exposed to each group for 14 days. Visualized colonies were photographed. The data were obtained from three independent experiments performed in triplicate, and the representative photos were shown.

The increasing research on the antitumor activity of Sch A against a variety of cancer cells has aroused great interest in studying the potential of Sch A for CRC cells. Thus, the antiproliferative effects of Sch A (Figure 1A) on four CRC cell lines RKO, DLD-1 SW620, SW480 and normal cell line CCD 841 CoN were determined through MTS assay. As shown in Figure 1B, Sch A has the strongest and smallest inhibitory effect on RKO and DLD-1 cells, with an IC_50_ value of 68.65 μM and over 150 μM, respectively. In comparison, Sch A exhibited moderate inhibitory effects on SW620 and SW480 with 50% inhibition at 85.66 and 87.57 μM. Thus, RKO and SW620 cells were selected for the subsequent biological activity evaluation. Besides, Sch A exerted little effect on the cell viability of CCD 841 CoN cells, suggesting that Sch A possessed a favorable selective inhibition on CRC cells (Figure 1B).

Furthermore, colony-forming assays were performed to assess the long-term inhibitory effects of Sch A treatment. After 24 h of treatment with Sch A (75 and 150 μM), the cell culture was replaced with fresh medium every 3 days. On the 14th day, cell colonies were stained with 0.1% Crystal Violet and photographed. Compared with the negative control, Sch A treatment produced a stronger inhibitory effect on cancer cell growth in a dose-dependent manner (Figure 1C), which is highly consistent with the results of the MTS analysis. All these data suggest that Sch A effectively inhibited the growth of multiple CRC cell lines.

### Sch A induces CRC cell cycle arrest

**Figure 2:**
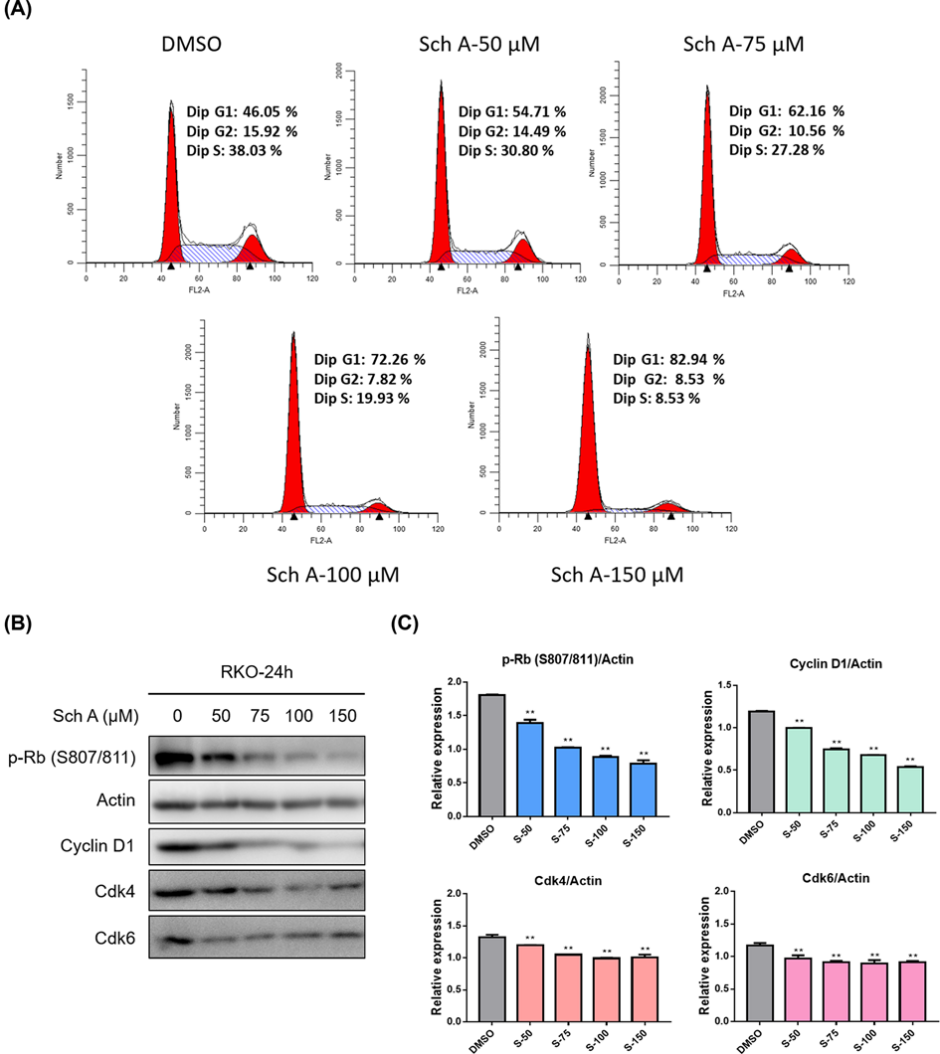
Sch A induced the cell cycle arrest of RKO cells (**A**) RKO cells were treated with Sch A (50, 75, 100, and 150 μM) for 24 h, and the cell cycle distribution was analyzed by flow cytometry (Becton Dickinson FACSCalibor, BD Biosciences, Franklin Lakes, NJ). The representative histogram of the cell cycle distribution was shown. Data are presented as means ± SDs of three independent experiments conducted in triplicate. (**B**) Western blot analysis of cell cycle-related protein p-Rb (S807/811), Cyclin D1, Cdk4, and Cdk6. Actin was shown as the control of equal loading. (**C**) The relative expression of cell cycle-related proteins (p-Rb (S807/811)/Actin, Cyclin D1/Actin, Cdk-4/Actin, and Cdk-6/Actin) was quantified using the ImageJ software and analyzed by GraphPad Prism 7. **P<0.01 compared with negative control.

To clarify whether the anti-growth effect of Sch A involved cell cycle arrest, the proportion of cells in G_0_/G_1_, S, and G_2_/M phases were counted by flow cytometry. The results demonstrated that Sch A effectively arrested RKO cells at the G_0_/G_1_ phase (Figure 2A). On the other hand, Western blot results demonstrated that Sch A synergistically acted on the expression of G_0_–G_1_ transition related proteins in a dose-dependent manner, such as phosphorylated Rb, Cyclin D1, Cdk4, and Cdk6 (Figure 2B,C). These results suggest that Sch A significantly arrested the cells in the G_0_/G_1_ phase by reducing the expression of cell cycle-related proteins.

### Sch A promotes CRC cells apoptosis

**Figure 3:**
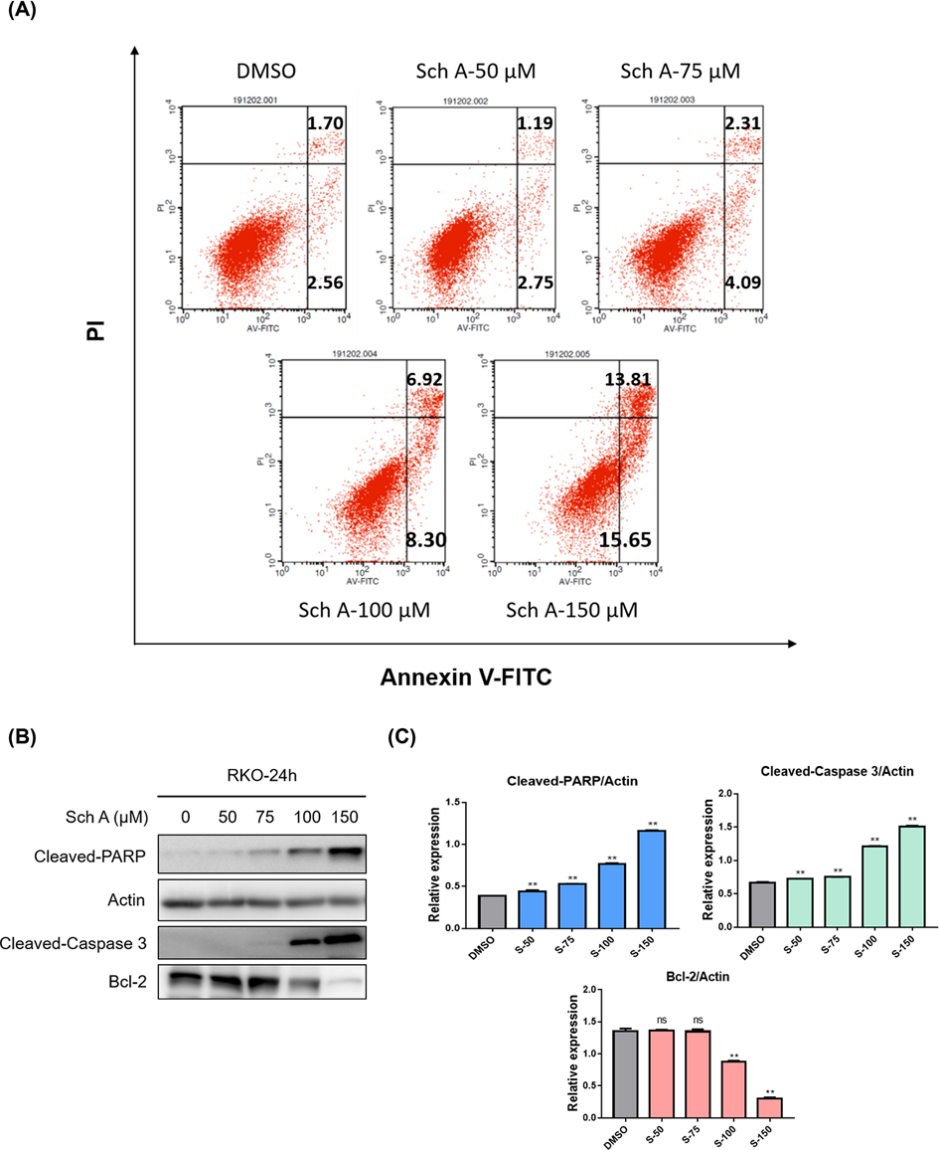
Sch A promoted RKO cells apoptosis (**A**) RKO cells were treated with Sch A (50, 75, 100 and 150 μM) for 24 h, apoptosis was assessed by Annexin V/ PI staining. Data are presented as means ± SDs of three independent experiments conducted in triplicate. (**B**) Total protein was extracted and the expression of Cleaved-PARP, Cleaved-Caspase 3 and Bcl-2 was examined by Western blot. Actin was shown as the control of equal loading. (**C**) The relative expression of apoptosis-related proteins (Cleaved-PARP/Actin, Cleaved-Caspase 3/Actin and Bcl-2/Actin) was quantified using the ImageJ software and analyzed by GraphPad Prism 7. **P<0.01 compared with negative control.

Annexin V-FITC/PI double staining assay was performed to determine whether Sch A exerted pro-apoptotic effects on CRC cells. The flow cytometry results showed that the early- and late-stage apoptosis rates of RKO cells were significantly increased after Sch A treatment for 24 h (Figure 3A). To elucidate the mechanisms of Sch A-induced pro-apoptosis effects, we further analyzed the expression levels of the apoptosis-related proteins Cleaved-caspase-3, Cleaved-PARP, and Bcl-2 by Western blotting. As shown in Figure 3B,C, the expression of pro-apoptotic molecules (Cleaved-PARP and Cleaved-caspase-3) was significantly elevated, while the anti-apoptotic protein Bcl-2 was decreased.

### Sch A binds directly to HSF1 and inhibits heat shock-induced HSF1 activation

Accumulating evidence shows that HSF1 exerted strikingly multifaceted effects on the carcinogenesis process of a variety of hematologic and solid tumor malignancies (e.g., T-cell acute lymphoblastic leukemia, chronic lymphocytic leukemia, breast cancer, gastric cancer, CRC, and so on). The abnormal expression and activation of HSF1 is not only closely related to poor clinical prognosis, but is also involved in a network of pro-oncogenic events, including cancer cell proliferation, survival, apoptosis resistance, and glucose metabolism. Previously, Li et al. reported that HSF1 epigenetically altered the expression of GLS1 and its downstream mTOR activation, thereby accelerating colorectal carcinogenesis [33]. Besides, Mendillo et al. [13] demonstrated that HSF1 drove a transcriptional program, which strongly promoted the survival and proliferation of CRC cells. Considering that the molecular mechanism of Sch A has not been fully elucidated, we speculate that the anti-CRC effects of Sch A might be through inhibition of HSF1 activation.

**Figure 4:**
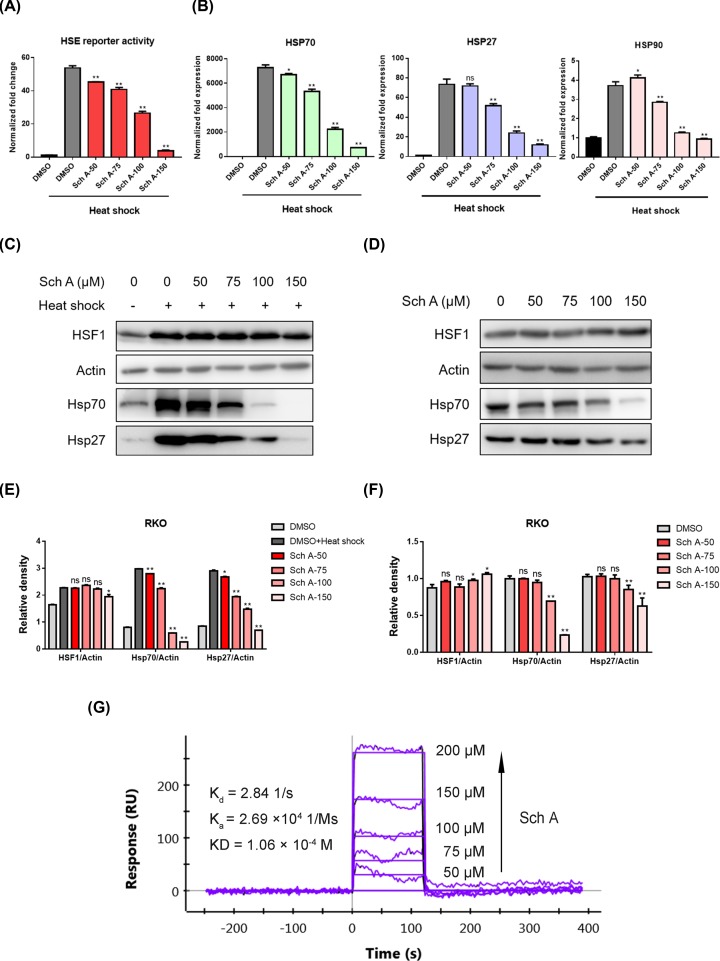
Sch A directly bound to HSF1, suppressed heat shock-induced HSE reporter activity and the mRNA transcription and protein expression of HSF1 downstream genes HSP70, HSP27, and HSP90 under both heat shock and basal conditions (**A**) RKO cells expressing the p(HSE)_4_-Basic reporter were pretreated for 30 min with the indicated concentrations of Sch A and then exposed to heat shock at 43°C for 1 h with 5-h recovery at 37°C. Luciferase activity was determined with a Dual-Glo® Luciferase Assay System (Promega, U.S.A.). Each experiment was repeated three times, and each value is the mean ± SD. Group comparisons were performed using one-way ANOVA with post-hoc Dunnett’s test (*P<0.05, **P<0.01). (**B,C**) After RKO cells were treated with Sch A (50, 75, 100, and 150 μM) at 37°C for 30 min, exposed to heat shock at 43°C for 1 h, and then incubated at 37°C for 30 min; the quantitative analysis of mRNA levels of HSP70, HSP27, and HSP90 was performed using quantitative real-time PCR, and the protein expression of HSF1, Hsp70, and Hsp27 was determined by Western blot. (**D**) RKO cells were treated with Sch A (50, 75, 100, and 150 μM) for 24 h, and whole cell lysates were extracted and analyzed by Western blot. (**E,F**) The relative expression of HSF1 and its downstream signaling proteins (HSF1/Actin, Hsp70 3/Actin, and Hsp27/Actin) was quantified using the ImageJ software and analyzed by GraphPad Prism 7. *P<0.05, **P<0.01 compared with negative control. (**G**) Direct-binding affinity between Sch A and HSF1 was demonstrated by SPR. Abbreviations: *K*_a_, association constant; *K*_d_, disocciation constant; KD, equilibrium dissociation constant.

First, we determined the inhibitory effects of Sch A on the HSF1 luciferase reporter. Surprisingly, we found Sch A indeed suppressed heat shock-induced HSF1 reporter activity in a concentration-dependent manner, and displayed approximately 50% inhibition at 100 μM (Figure 4A). To confirm this inhibitory effect, we evaluated the effect of Sch A on the expression of HSF1 downstream target genes. The mRNA and protein levels of HSP70, HSP90 and HSP27 were analyzed by qRT-PCR and Western blot, respectively. Because the time required for mRNA transcription is much less than that for protein translation, we measured the mRNA levels and protein expressions at two different time points (the RNA was isolated after recovery at 37°C for 0.5 h, and recovered for 5 h for protein extraction) based on previous research [34]. As shown in Figure 4B, Sch A effectively blocked the transcription of HSF1 target genes (*HSP70, HSP27*, and *HSP90*). The results (Figure 4C–F) further demonstrated that treatment of Sch A effectively down-regulated HSF1-regulated downstream signaling activation induced by heat shock, as well as the basal expression of Hsp 70 and Hsp 27 in RKO cells. Based on these results, it is suggested that the mechanism of Sch A’s anti-CRC effects might be through inhibition of HSF1 signal activation.

To investigate whether Sch A directly binds to HSF1, SPR experiments were performed. As shown in Figure 4G, the SPR response value increased gradually with elevated Sch A concentrations and a moderate equilibrium dissociation constant (KD) of 106 μM was determined. Collectively, these results indicate that Sch A binds directly to HSF1, thereby preventing the activation of HSF1 under stress and basal conditions, as well as subsequent transcription and expression of HSF1 target genes.

### Analysis the binding mode between HSF-1 and Sch A

**Figure 5:**
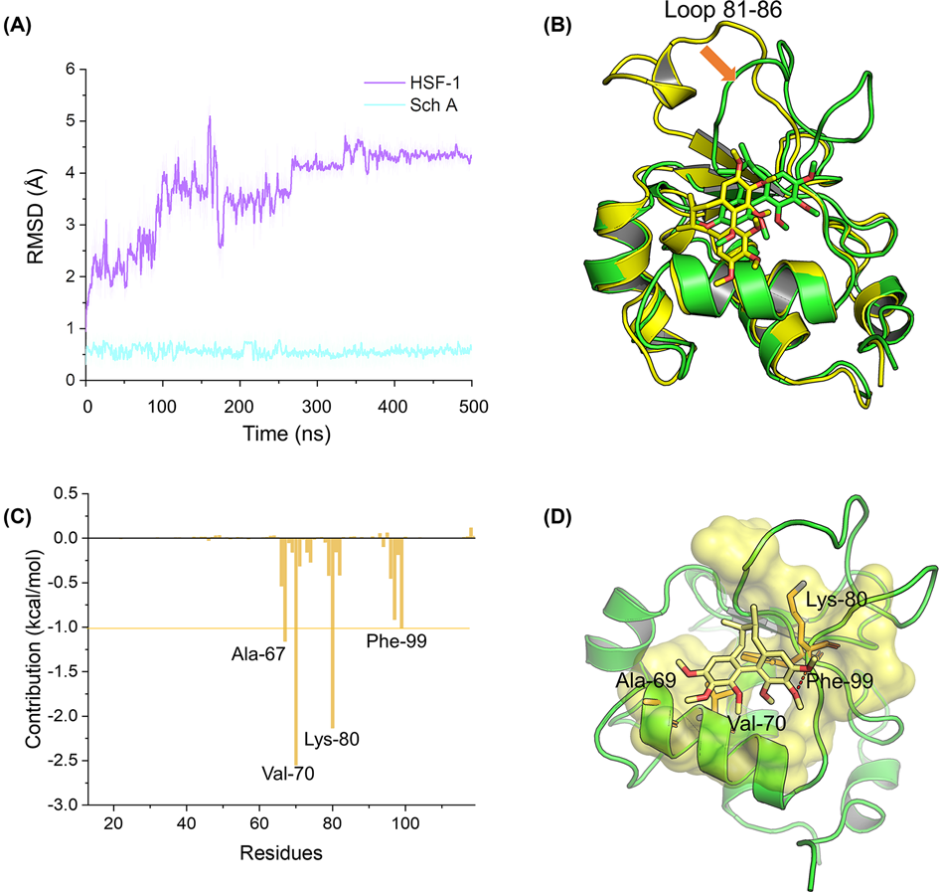
Molecular modeling analysis of Sch A to the binding pocket of HSF-1 (**A**) RMSD curves for the 500 ns MD simulation. (**B**) Alignment of the initial structure from molecular docking and the last snapshots from MD simulation. (**C**) Per-residue contribution calculated by MM/GBSA method. (**D**) Structural analysis of the key residues of Sch A to HSF-1.

To further elucidate the possible binding mode between HSF-1 and Sch A, molecular docking was performed to predict the interaction between HSF-1 and Sch A. Then, a stable MD trajectory was obtained by applying 500 ns MD simulations. First, the root-mean square deviations (RMSDs) of the HSF-1 protein backbone atoms and the Sch A heavy atoms were monitored to analyze the stability of the simulated system. As shown in Figure 5A, HSF-1 exhibited small fluctuations within 1 Å after 270 ns. In the whole MD simulation, Sch A was relatively stable, and the RMSD fluctuated within 1 Å. Therefore, the obtained stable trajectory was used for further analysis. Alignment of the initial structure from molecular docking with the last snapshots from MD simulation showed quite similar results (Figure 5B). However, the loop with residues numbered from 81 to 86 showed large conformational changes to accommodate Sch A binding (Figure 5B). The above results indicated that a series of reasonable conformations for HSF-1/Sch A were obtained through 500 ns MD simulation. These findings suggest that the role of individual residues in protein–ligand recognition patterns was determined by using the last 100 ns simulation trajectory. As shown in Figure 5C, the most contributing residues were Val^70^, Lys^80^, Ala^67^, and Phe^99^. Structural analysis indicated that Lys^80^ formed a key hydrogen bond with Sch A, and that the other predominant residues were hydrophobic amino acids (Figure 5D). These findings suggest that hydrogen bonding and non-polar interactions may contribute to the binding of Sch A to HSF-1.

## Discussion

HSF1 is an emerging cancer therapeutic target in recent research. The human HSF family comprises six members: HSF1, HSF2, HSF4, HSF5, HSFX, and HSFY, among which HSF1 is the most studied member and plays a dominant role in heat shock response [9]. The activation process of HSF1 could be simply divided into three steps: stress sensing, trimerization, and post-translational modifications (PTMs) [12]. In response to various stresses such as heat shock, oxidants, and proteotoxic agents, the monomeric, inactive HSF1 oligomerizes, translocates into the nucleus, and then binds to the HSE and drives the transcriptional activation of target genes. Subsequently, HSF1 is modified by several PTMs, such as phosphorylation, acetylation, and SUMOylation, which decides the ultimate fate of HSF1—to continuously support the transcription, degradation or recycling of target genes into the cytoplasm. For a long time in the past, researchers have agreed that the main biological function of HSF1 is to specifically turn on downstream HSP expression and then promote cancer cells to maintain a stable state of cellular proteins. However, as research progresses, some exciting results suggest that HSF-1 does play a broader and more profound role in tumorigenesis. Dai et al. [35] reported that HSF1 is a key factor supporting the oncogenic transformation of normal cell lines induced by RAS oncogene mutations or hotspot mutations in tumor suppressor p53, and the survival rate of malignant transformed cells is more dependent on HSF1 expression than non-transformed cells. Another stimulating result by Mendillo et al. [13] further elaborated that the HSF1-regulated genes form a comprehensive and coordinated pro-oncogenic signal network, including cell cycle, apoptosis, DNA repair, and energy metabolism, far beyond its conventional response to heat stress. Besides, this study also pointed out that the HSF1-regulated transcriptional targets were hyperactivated in a wide range of human malignancies and possessed potential prognostic value, especially in breast cancer and colon cancer. In many recent studies, researcher also demonstreated that HSF1 was universally overexpressed in a majority of CRC cell lines, and the growth of CRC cells was severely restraint by chemical inhibition or genetic ablation of HSF1. In general, HSF1, as a pleiotropic effect transcription factor, has selected many downstream molecules to participate in maintaining the proteomic stability of cancer cells and generate ‘Non-oncogene addiction’ of cancer. All the above evidence suggests that HSF1 should be considered as an attractive target for CRC therapeutics.

During the past few years, some progress has been made in the discovery of effective HSF1 inhibitors through high-throughput screening of synthetic compounds [36]. Two research groups successively reported that KRIBB11 and I_HSF_115 both directly interact with HSF1, and effectively inhibit heat shock-induced HSF1 reporter activity in a concentration-dependent manner, with IC_50_ values of 1.2 and 0.7 μM, respectively [34,37]. However, some recent evidence suggests that both KRIBB11 and I_HSF_115 display some off-target effects. On the other hand, the search for HSF1 inhibitors derived from natural products is also receiving widespread attention [38]. Two dietary flavonoid compounds (quercetin and fisetin) were identified as the inhibitors of the heat shock response and the expression of HSF1 downstream target genes (*Hsp70, Hsp27*, and *BAG*3). Nonetheless, there is still no compelling evidence for a direct interaction between HSF1 with quercetin or fisetin. In addition, many other studies have also revealed that quercetin and fisetin might simultaneously inhibit various signaling pathways, such as PI3K/Akt and MAPK pathways, suggesting that they should not be considered as specific HSF1 inhibitors [12]. Therefore, the discovery of novel effective and selective direct inhibitors of HSF-1 remains an urgent and necessary task, which is essential to improving HSF1 as a therapeutic target.

In this research, we mainly investigated the anti-CRC activity of Sch A and explored its underlying molecular mechanism. The antitumor effects of Sch A were well-studied in several recent investigations. As a major member of dibenzocyclooctadiene lignans, Sch A not only impaired the proliferation, migration, and invasion of breast cancer, ovarian cancer, and thyroid cancer through prompting ER stress, inactivating Akt signals, and down-regulating miR-429, but also acted as a favorable chemosensitization agent in breast cancer and colon carcinoma [18,19,39,40]. Moreover, Xian et al. [41] surprisingly found that Sch A could even improve the gefitinib resistance in non-small cell lung cancer cells (NSCLC) via inhibition of IKKβ/NF-κB signaling. However, the inhibitory effects of Sch A against some other cancers, such as CRC, has not been fully evaluated. Herein, we demonstrated that Sch A selectively inhibited the proliferation of CRC cell lines with little effect on normal colonic epithelial cells. The results of the clone formation analysis further indicated that Sch A has a long-term inhibitory effect on CRC cells. Through flow cytometry and Western blot, our data suggested that Sch A suppressed the growth of CRC cells via induction of cell cycle arrest and apoptosis. By determining the inhibitory effect of Sch A on luciferase reporter activity and HSF1 target genes expression, we found Sch A dose-dependently reduced heat shock-induced HSF1 activation in CRC cells. Notably, even under rest conditions, Sch A treatment also down-regulated the expression of HSF1 target genes (*HSP70* and *HSP27*), resulting in continuous inhibition of basal HSF1 activity, which may be more effective in improving the viability of cancer cells because heat shock stimuli was not a common event existing in all types of cancer. Based on the key role of HSF1 in maintaining the proliferation and survival of CRC cells and preventing apoptosis, and the consistency of the anti-Sch A anti-growth activity and HSF1 inhibitory effect, we speculate that the anti-CRC effects of Sch A might be mainly mediated via inhibition of HSF1. Finally, the SPR results showed a concentration-dependent interaction between HSF1 and Sch A, suggesting that Sch A is a direct HSF1 inhibitor. In order to better clarify the anti-CRC effect of Sch A *in vivo* and the relationship between Sch A’s biological activity and HSF1, related animal experiments are currently being performed.

HSF1 is structurally connected by five conserved domains as indicated: the DNA-binding domain (DBD), the heptad repeat oligomerization domain (HR-A/B), a regulatory domain (RegD), an oligomerization repressing domain (HR-C) and a carboxyl-terminal transactivation domain (TAD) [20]. Although some excellent studies have identified a number of potent HSF1 inhibitors, such as KRIBB11 and I_HSF_115, the molecular interaction mode between HSF1 and these inhibitors has not been studied, which seriously hinders their further medicinal chemistry rational modification. Therefore, we also performed molecular docking and dynamic simulations to analyze the basic mode of interaction and the key binding sites between Sch A and HSF1. Our molecular docking and simulation analyses showed that Sch A bound to HSF1 Lys^80^ throuugh hydrogen bonding, and interacted with Val^70^, Ala^67^, and Phe^99^ through hydrophobic interactions. Since Sch A’s chemical structure lacks hydrogen bonding donors, such as carbonyl group and nitrogen atoms, the binding affinity between HSF1 and Sch A remains unsatisfactory. Based on the above structural information, we plan to add more hydrogen-bonded donor groups asymmetrically to the two benzenes to enhance their target affinity. Beyond that, overcoming the low water solubility of Sch A has always been one of the most important issues in improving its druggability. Introducing a hydrophilic side chain into the chiral methyl of Sch A far from the interaction interface of HSF1/Sch A may effectively improve its poor solubility and its inhibitory effect on HSF1. Furthermore, it has been reported that the acetylation status of Lys^80^ within the HSF1 DBD strongly impacts its DNA-binding activity, suggesting that Lys^80^ in HSF1 DBD could be a potential target site for the development of highly selective HSF1 inhibitors [42]. Thus, our next modification work will focus on this interesting area.

## Conclusions

To sum up, Sch A significantly inhibited the growth of CRC cells by inducing cell cycle arrest and apoptosis. Mechanistically, our results demonstrated that Sch A could bind directly to HSF1, thereby decreasing heat shock-induced HSF1 target gene expression and inhibiting basal activation of HSF1 signaling. Finally, molecular docking and molecular simulations of the Sch A/HSF1 complex suggested that Sch A formed key hydrogen bond and hydrophobic interaction with HSF1. Overall, this work provides a novel natural-derived HSF1 inhibitor for CRC treatment and some useful information to guide the rational modification of Sch A in medicinal chemistry.

## Abbreviations

CRC: colorectal cancer
DBD: DNA-binding domain
EGFR: Epidermal growth factor receptor
ER: Endoplasmic reticulum
GLS1: Glutaminase 1
IKKβ: IkappaB Kinase β
HRP: horseradish peroxidase
HSE: heat-shock element
HSF1: heat shock factor 1
HSP: heat-shock protein
MD: molecular dynamics
MM/GBSA: Molecular Mechanics/Generalized Born Surface Area
PARP: poly ADP-ribose polymerase
PI: propidium iodide
PSR: proteotoxic stress response
PTM: post-translational modification
RMSD: root-mean square deviation
Sch A: Schizandrin A
SPR: surface plasmon resonance
